# Exploring Methacrylated Gellan Gum 3D Bioprinted Patches Loaded with Tannic Acid or L-Ascorbic Acid as Potential Platform for Wound Dressing Application

**DOI:** 10.3390/gels11010040

**Published:** 2025-01-05

**Authors:** Federica Scalia, Alessandra Maria Vitale, Domiziana Picone, Noemi De Cesare, Maria Swiontek Brzezinska, Beata Kaczmarek-Szczepanska, Alfredo Ronca, Barbara Zavan, Fabio Bucchieri, Marta Anna Szychlinska, Ugo D’Amora

**Affiliations:** 1 Department of Biomedicine, Neuroscience and Advanced Diagnostics (BIND), University of Palermo, 90127 Palermo, Italy; federica.scalia02@unipa.it (F.S.); alessandramaria.vitale@unipa.it (A.M.V.); domiziana.picone@unipa.it (D.P.); fabio.bucchieri@unipa.it (F.B.); 2 Institute of Polymers, Composites and Biomaterials (IPCB), National Research Council (CNR), 80125 Naples, Italy; noemidecesare@cnr.it (N.D.C.); alfredo.ronca@cnr.it (A.R.); 3 Department of Environmental Microbiology and Biotechnology, Faculty of Biological and Veterinary Sciences, Nicolaus Copernicus University in Torun, Lwowska 1, 87-100 Torun, Poland; swiontek@umk.pl; 4 Department of Biomaterials and Cosmetics Chemistry, Faculty of Chemistry, Nicolaus Copernicus University in Torun, Gagarin 7, 87-100 Torun, Poland; beata.kaczmarek@umk.pl; 5 Department of Translational Medicine, University of Ferrara, 44121 Ferrara, Italy; barbara.zavan@unife.it; 6 Department of Precision Medicine in Medical, Surgical and Critical Care (MEPRECC), University of Palermo, 90127 Palermo, Italy; martaanna.szychlinska@unipa.it

**Keywords:** methacrylated gellan gum patches, tannic acid, L-ascorbic acid, 3D bioprinting, wound healing

## Abstract

To improve wound healing, advanced biofabrication techniques are proposed here to develop customized wound patches to release bioactive agents targeting cell function in a controlled manner. Three-dimensional (3D) bioprinted “smart” patches, based on methacrylated gellan gum (GGMA), loaded with tannic acid (TA) or L-ascorbic acid (AA) have been manufactured. To improve stability and degradation time, gellan gum (GG) was chemically modified by grafting methacrylic moieties on the polysaccharide backbone. GGMA patches were characterized through physicochemical, morphological and mechanical evaluation. Kinetics release and antioxidant potential of TA and AA as well as antimicrobial activity against common pathogens *Pseudomonas aeruginosa*, *Staphylococcus aureus* and *Escherichia coli* in accordance with ISO 22196:2011 are reported. The cytocompatibility of the patches was demonstrated by direct and indirect tests on human dermal fibroblasts (HDF) as per ISO 10993. The positive effect of GGMA patches on cell migration was assessed through a wound healing assay. The results highlighted that the patches are cytocompatible, speed up wound healing and can swell upon contact with the hydration medium and release TA and AA in a controlled way. Overall, the TA- and AA-loaded GGMA patches demonstrated suitable mechanical features; no cytotoxicity; and antioxidant, antimicrobial and wound healing properties, showing satisfactory potential for wound dressing applications.

## 1. Introduction

Skin wound healing is an intricate physiological process in response to tissue injury. It is a complex collaboration of different cell types and tissues, cytokines and mediators [1]. It requires high coordination between several biological events such as homeostasis, cell proliferation and migration, extracellular matrix (ECM) deposition and remodeling, along with inflammation, angiogenesis, thrombosis and re-epithelialization [2]. This spatial and temporal organization ensures that the wound heals as quickly as possible to prevent further damage or infection. The alteration of these physiological events can lead to impaired healing with the formation of chronic wounds and hypertrophic scars or keloids [2]. Many factors can interfere with the healing process, such as bacterial colonization, hypoxic environment, altered collagen synthesis and impaired cellular response. The latter may be due to systemic diseases, such as metabolic syndrome, and/or chronic pathologic conditions, such as malnutrition, alcoholism, smoking or other factors such as pressure, infection, tissue edema and dehydration [2].

Recently, bioengineered personalized therapeutic methods have been developed to accelerate wound healing, protect against infections, enhance cellular response and promote collagen synthesis. In this scenario, the three-dimensional (3D) bioprinting technique represents a promising method for manufacturing functionalized wound-healing patches as it offers the ability to combine biopolymers and biomolecules with therapeutic potential [3]. The advantage of 3D printing technology in the fabrication of wound dressing permits the manufacturing of custom-made 3D structures with controlled internal micro-architecture that matches the skin defect [4]. For this purpose, biomaterials play a pivotal role since they act exactly as an ECM template with the main aim of restoring damaged tissue and re-establishing its physiological function. Due to their high biocompatibility and structural resemblance to native source tissue, many natural biomaterials have been used for treating skin wounds [5]. Among these, hydrogels represent an important class of bioinks for skin tissue engineering applications [6]. Hydrogel-based scaffolds derived from natural polymers, such as polysaccharides, are widely used due to their ability to mimic the natural tissue structure. Moreover, they exhibit the potential to be encapsulated with bioactive compounds and permit controlled drug delivery, making them particularly attractive for smart wound dressing manufacturing. However, despite these important properties, hydrogels’ drawback is poor mechanical stability limiting their use in 3D bioprinting, and for this reason, they need to be properly chemically functionalized [7,8].

Among the polysaccharides, gellan gum (GG) presents a wide-range perspective of applications due to its interesting intrinsic physicochemical features; high stability; and biodegradable, biocompatible and sustainable features [9,10]. GG is a linear negatively charged exopolysaccharide, produced by *Sphingomonas* bacteria. It is characterized by the presence of four repeating carbohydrates in the main chain: two D-glucose carbohydrates, one D-glucuronic acid and one L-rhamnose. At low temperatures, GG is in the form of a double helix; meanwhile, at high temperatures, it has the form of random coils [11]. With encouraging results in wound dressing applications, GG has recently been the subject of extensive research [11]. GG scaffolds/membranes/patches exhibit excellent cytocompatibility [12] and moderate water vapor transmission rate values that are appropriate to keep the wound bed moist for an accelerated wound healing process [13]. By combining GG with other natural/synthetic polymers, or by using crosslinking agents, it is possible to tune the mechanical properties, producing excellent scaffolds/patches that can be easily handled and applied as wound dressing [14,15]. One of the limitations of the use of GG-based wound dressing for infected wounds is that they do not show antibacterial properties toward Gram-positive and Gram-negative bacterial strains. However, this limitation can be overcome by loading GG-based hydrogels with different bioactive agents, such as antibiotics and essential oils [16,17]. Furthermore, physically crosslinked GG hydrogels can degrade in a short period of time because of their instability in physiological conditions due to the exchange of divalent cations with monovalent ones. This aspect is of paramount importance since the patch should not completely degrade before the proliferation phase of wound healing, which typically lasts two to four weeks, is finished [18]. To address this, covalent crosslinking, using chemical crosslinkers, can enhance the mechanical and stability characteristics of GG. Photopolymerization has indeed gained significant interest as an alternative method for creating methacrylated GG (GGMA) hydrogels. This technique involves using UV light to allow crosslinking, leading to improved structural and mechanical integrity [8]. L-ascorbic Acid (AA), or Vitamin C, has been widely investigated for its positive effects on wound healing as its amount is scarce in the injured tissue [19]. AA plays a crucial role in the synthesis and post-translational modification of collagen, increasing the tensile strength and leading to better wound closure [20,21]. Moreover, it has antioxidant properties and aids in reducing tissue damage, accelerating the wound healing process. AA also plays an importantrole in immunity, fundamental in wound protection against infections. Furthermore, it was shown to display anti-inflammatory potential and support the synthesis of pro-angiogenic biomolecules that ultimately contribute to wound healing [22].

Another promising biomolecule, explored for wound healing promotion, is represented by tannic acid (TA). TA is a plant polyphenol derived from *Rhus* and *Quercus* species, and it is considered an antioxidant, antiviral, anti-inflammatory and antimicrobial agent [23,24,25,26].

Due to their multifactorial properties and positive effects on the wound healing process, AA and TA were considered here as promising bioactive compounds used to load bioprinted GGMA patches for wound healing application. Moreover, their effect on the physicochemical, morphological, mechanical, in vitro and antimicrobial properties of the GGMA patches were fully investigated.

## 2. Results and Discussion

Wound healing treatment represents a persistent challenge in clinical practice. Severe trauma can cause permanent harm and life-threatening circumstances. The current dressings present several limitations such as poor adherence to the wound bed, lack of mechanical support, instability and lack of biological activity. In this scenario, great advancements have been made in the design of innovative smart wound dressings, able to adapt to the shape of the wound site, ensuring their customization, and able to release biological cues to target cells function in a controlled way [3,27]. To this end, biofabrication techniques have revolutionized wound management. Indeed, traditional fabricated hydrogel patches do not present an adequate porous structure for close-fitting integration, while 3D printing can easily produce highly porous hydrogel-based structures [28]. Therefore, the present work aimed at manufacturing 3D bioprinted smart patches, based on GGMA, loaded with TA and AA. To improve stability as well as the degradation time, GG was chemically modified to graft methacrylic moieties on the polysaccharide backbone.

### 2.1. Physicochemical Characterization of the Bioink

**Figure 1:**
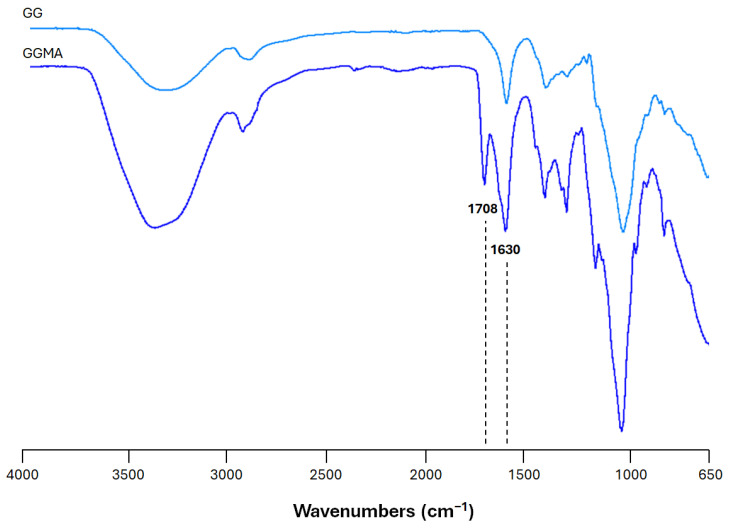
ATR-FTIR spectra of gellan gum (GG) and methacrylated GG (GGMA) between 4000 and 650 cm^−1^.

Fourier transform infrared (ATR-FTIR) spectroscopy was employed to assess the methacrylation of GG. The ATR-FTIR spectra of unmodified GG and GGMA are shown in Figure 1. As is common for all polysaccharides, both spectra show skeletal vibration involving the C–O stretching band at 1030 cm^−1^ and a large −OH stretching band above 3000 cm^−1^. Furthermore, it is possible to observe the characteristic peaks at 1120 and 1300–1470 cm^−1^ related to C–C stretching and C–H bending, respectively. The peaks at 1630 cm^−1^ and 1708 cm^−1^, ascribable to the C=C stretching typical of the methacrylic moieties in the GG double bond and the carbonyl (C=O) stretching, confirm the chemical modification.

### 2.2. Property of Hydrogel Patches

3D bioprinted patches were manufactured and loaded with TA and AA by passive diffusion of both bioactive agents into the polymer matrix. ATR-FTIR spectroscopy was performed to assess the presence of all the compounds. 

**Figure 2:**
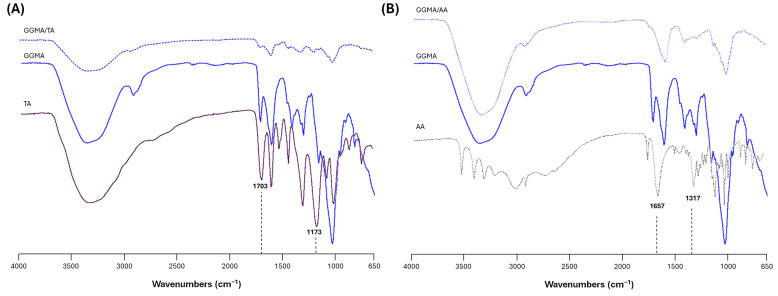
ATR-FTIR spectra of (**A**) tannic acid (TA), GGMA and GGMA/TA and (**B**) L-ascorbic acid (AA), GGMA and GGMA/AA between 4000 and 650 cm^−1^.

Figure 2A shows the typical spectrum of TA characterized by a strong band between 3600 and 3000 cm^−1^, due to hydroxyl groups (O-H), H bonds and C-H (stretching vibrations of aromatic medium). A band due to the alkane medium (C-H) can be noticed at around 2800 cm^−1^. The characteristic bands of TA at 1703 cm^−1^ (C=O stretching) and 1173 cm^−1^ (C-O) are correlated to the aromatic esters [29]. By comparing the different spectra, the presence of bands in GGMA/TA at 1536 cm^−1^, 1192 cm^−1^ and 1077 cm^−1^ were consistent with that of TA, indicating the successful loading of TA into the 3D patches [30]. However, it is widely reported that TA has the ability to form hydrogen bonds with the chemical moieties found in different biopolymers such as collagen or chitosan [31].

The ATR-FTIR spectrum of neat AA is reported in Figure 2B. The spectrum revealed the presence of the bands related to the stretching vibration of the C-C double bond and of enol-hydroxyl at 1657 cm^−1^ and 1317 cm^−1^, respectively [32]. By comparing the spectra, it is possible to observe that the band centered at 1708 cm^−1^ (GGMA) shifted to 1746 cm^−1^ (GGMA/AA). Meanwhile, for the other bands, interpretation is hard due to their overlapping [32]. Similarly, in the case of L-ascorbic acid, a possible interaction based on hydrogen bonds could also be hypothesized.

**Figure 3:**
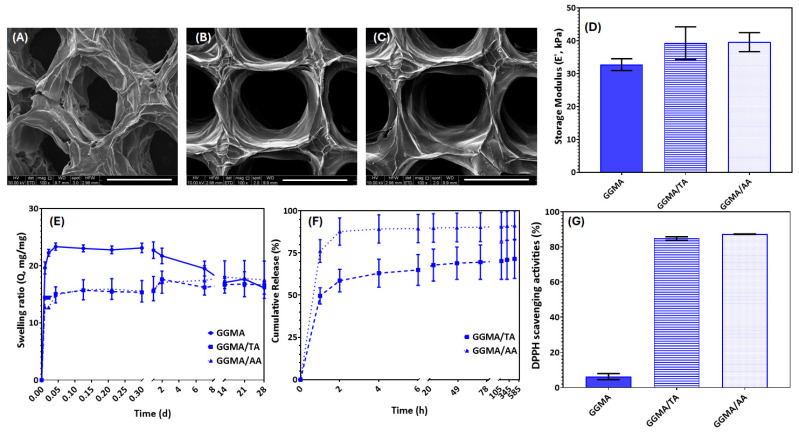
Morphological, mechanical and physicochemical characterization. Scanning electron microscopy (SEM) images of (**A**) GGMA, (**B**) GGMA/TA, (**C**) GGMA/AA. Scale bars: 1 mm. High vacuum 10.00 kV, magnification 100×. (**D**) Storage modulus of bulk GGMA, GGMA/TA and GGMA/AA materials; (**E**) swelling behavior and (**F**) cumulative release of TA and AA from GGMA-based patches. ◊ with solid line indicates GGMA, □ with dashed line indicates GGMA/TA, Δ with dotted line indicates GGMA/AA. (**G**) 2,2-diphenyl-1-picrylhydrazyl (DPPH) scavenging activities of GGMA, GGMA/TA and GGMA/AA. Data reported as mean value ± standard deviation (S.D.), n = 6.

Porous hydrogel patches were successfully manufactured by 3D bioprinting (Figure S1). A passive diffusion filling approach was adopted to enrich GGMA patches with TA and AA, instead of functionalizing the bioink before, to overcome specific disadvantages during the printing process. First, from a chemical point of view, L-ascorbic acid is unstable in water solution [33] and the use of a bioink could affect its potential as a bioactive component. Furthermore, as already demonstrated, TA and AA slightly acted as crosslinker agents. For this reason, the functionalized bioinks were not homogeneous compared to GGMA alone, influencing the printing process. Furthermore, as pre-crosslinked bioink, the fibers were not continuously deposited, affecting the overall stability of the structure. After the manufacture, the three patches (GGMA, GGMA/TA and GGMA/AA) were characterized from a morphological point of view after being freeze-dried because they were intended to be placed onto the wound surface following lyophilization. As it is possible to observe from scanning electron microscopy (SEM) images reported in Figure 3A–C, all the patches showed a microporosity of 100% open and interconnected, which represents a key property for wound healing application, providing a viable microenvironment for cell adhesion, migration and proliferation [34]. Indeed, cells should be able to colonize the structure, allowing the flux of metabolites, O_2_ and waste [35]. However, qualitatively, after passive diffusion of TA and AA, the fibers’ patches appeared more compact and denser, as a result of both acids’ interaction with the polymer matrix. For this reason, the pores appear slightly larger than the control.

As a wound dressing or patch, the materials should have adequate mechanical properties allowing them to support external forces without altering their structural integrity. It is widely known that skin has a Young’s modulus in the range of 10–50 kPa [36]. Previous studies suggested that regeneration is enhanced if the bulk modulus of the scaffold fits in this range, exceeding that of host dermal tissue, and the structure is able to persist throughout all stages of the remodeling phases [28,37]. Here, the mechanical behavior of bulk samples was characterized using a universal testing machine in a wet state at 37 °C (Figure 3D). The results showed that the storage modulus of bulk materials increased after TA and AA loading, because of the interaction between TA and AA with the polymer matrix through the formation of hydrogen bonds, as also evidenced by ATR-FTIR.

One of the critical aspects of wound care management is ensuring the dressings are able to absorb the exudate, maintaining a moist environment [38]. To this end, the swelling behavior of patches was studied for up to 28 days in physiological conditions (pH 7.4 and 37 °C) (Figure 3E). The results showed that GGMA patches were stable in the first 2 days; meanwhile, GGMA/TA and GGMA/AA showed higher stability for up to 28 days, without mass loss. Freeze-dried GGMA patches, upon contact with the rehydration medium, were able to swell up to a Q value of about 20; meanwhile, the passive embedding of TA and AA reduced the swelling ratio because of a more crosslinked network. There was no significant difference between the kinetics of the three distinct kinds of 3D printed patches over time, meaning that they reached their equilibrium at approximately the same time (15 min). However, all the structures maintained their 3D lattice-like structure without collapsing over time, suggesting their suitability for skin tissue engineering.

The kinetics release profiles of TA and AA are reported in Figure 3F. As it is possible to observe, GGMA/TA and GGMA/AA showed a burst release of 49.6 ± 4.8% and 76.2 ± 6.8%, respectively, in the first hour, followed by a continuous release until 71.5% and 91.3%, respectively. This result agrees with the previous data, suggesting a stronger interaction between GGMA and TA compared to AA. Tannic acid and L-ascorbic acid release rates mainly depend on the structure of the polymer matrix where they are embedded. For example, when TA was incorporated within microfibrillated cellulose, a sustained release was achieved for up to a week, and the addition of glycerin slightly decreased the release rate [39]. In the case of tannic acid-loaded chitosan/arginine–glycine–aspartic acid (RGD)/alginate scaffolds, a burst release of 57.12 ± 3.21% was detected at 24 h, followed by a gradual steady release of 8.45 ± 1.98% per day up to 90% in 5 days [40]. Meanwhile, Leon and Rojas described GG-based films as AA carriers for food antioxidants and nutritional applications [41]. Vivcharenko et al. showed how to treat chronic wounds by using chitosan/agarose film to supply ascorbic acid in a regulated manner. They found that there was no discernible ascorbic acid release for additional time points after a high burst release that happened within three hours [42]. Ravi et al.’s investigation produced a similar outcome, demonstrating that a material based on poly (ethylene glycol) methacrylate-grafted GG could deliver ascorbic acid continuously over an extended length of time. Indeed, due to its anionic nature, this matrix showed pH sensitivity and pH-dependent release. This represented a great advantage because the pH is typically alkaline in chronic wounds, leading to higher release [43]. Similarly, in the work of Niu et al., at pH = 7.4, the release efficiency of L-ascorbic acid from sodium alginate/humic acid/konjac hydrogel reached 94.57% at around 10 h because sodium alginate hydrogel has a good release effect on the drug in an alkaline solution, and the gel is highly responsive to an alkaline pH environment [44].

For skin wound healing, materials exhibiting antioxidant activity showed a positive impact by inhibiting molecular oxidation and restoring the normal physiological level of reactive oxygen species (ROS) [45]. Matrix metalloproteinases (MMPs) are crucial for tissue remodeling. It has been shown that tannic acid inhibits the activity of MMP-2 and 9. Tannic acid has been identified as a substance that enhances wound healing and tissue remodeling activity [45]. Similarly, ROS can be neutralized also by L-ascorbic acid, a great antioxidant. Furthermore, AA plays a pivotal role in the deposition of collagen and other organic elements of the intracellular matrix of tissues, including skin. However, its poor bioavailability, fast degradation and instability are some of the characteristics that limit its use in the biomedical field [46]. Indeed, its low stability is a serious drawback. It is a molecule that is rapidly oxidized, particularly in aerobic environments and when exposed to light. It first breaks down reversibly to dehydroascorbic acid and then irreversibly to oxalic acid [47]. For this reason, research has been focused on its encapsulation into a polymer matrix as a valuable approach to improve its main limitations [47]. Herein, the antioxidant properties of GGMA-based patches were studied by the 2,2-diphenyl-1-picrylhydrazyl (DPPH) test (Figure 3G). GGMA/TA and GGMA/AA showed DPPH scavenging activity values of 84.8% and 87.3% without significant differences, higher than the GGMA ones (6.3%), highlighting their potential in modulating ROS production during the inflammatory phases of the wound healing process. This finding aligns with the results of a study by Lee et al., who demonstrated that the antioxidant properties of chitosan/tannic acid films could be ascribed to tannic acid owing to the large number of phenolic hydroxyl groups in TA. Similarly, the addition of tannic acid (10% wt) to chitosan/microfibrillated cellulose/glycerin gels promoted good antioxidant scavenging activity values, achieving ~75% due to the effective scavenging ability of its polyphenol [39]. Tannic acid also represented a valuable additive that provided antioxidant properties of hyaluronic acid-based materials [26]. Regarding AA, different works have highlighted that it is a major antioxidant in human blood at concentrations of 40–80 μM [40].

### 2.3. In Vitro Assays

To test the biological safety and cytocompatibility of the three GGMA-based patches (GGMA, GGMA/TA and GGMA/AA) according to ISO 10993-5 standards [48], direct and indirect biocompatibility tests were performed in vitro using an HDF cell line. In the direct test, the cells were incubated for 24 and 48 h with the equilibrated patches or without any patch normal conditions (control condition, untreated HDFs), while in the indirect test, the cells were treated with the patch-conditioned media only.

**Figure 4:**
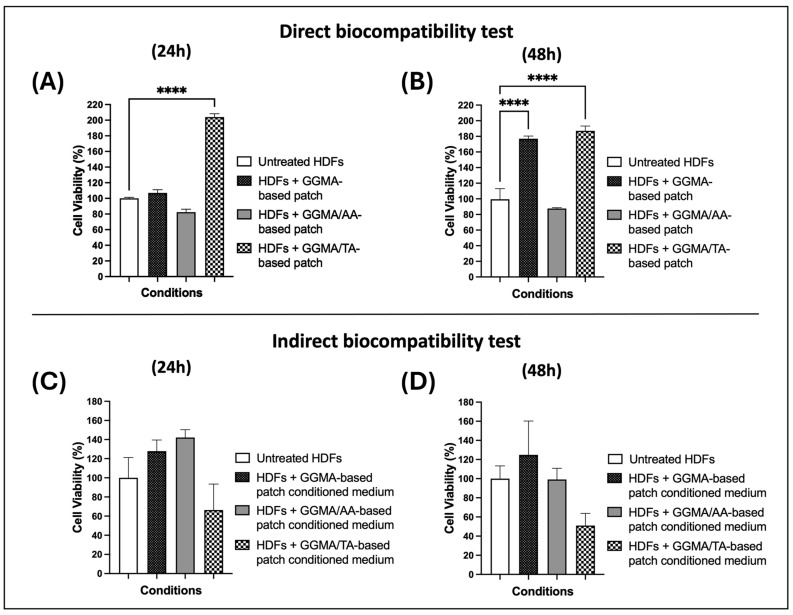
Histograms show direct and indirect biocompatibility tests. (**A**,**B**) Histograms show the percentage of viable cells in direct biocompatibility tests at 24 and 48 h, respectively. (**C**,**D**) Histograms show the percentage of viable cells in indirect biocompatibility tests at 24 and 48 h, respectively. White column: HDFs, untreated cells; black column: HDFs with GGMA patch (**A**,**B**) or GGMA-patch-conditioned medium (**C**,**D**); grey column: HDFs with GGMA/AA patch (A and B) or GGMA/AA-patch-conditioned medium (**C**,**D**); dotted column: HDFs with GGMA/TA patch (**A**,**B**) or GGMA/TA-patch-conditioned medium (**C**,**D**). Data, means ± standard S.D., were normalized to the untreated group mean. Two-way ANOVA and one-way ANOVA followed by Bonferroni’s test were performed for direct and indirect tests, respectively. **** *p* < 0.0001.

The direct test obtained results (Figure 4, A and B histograms) showed a significant increase in cell viability both at 24 h (204%) and 48 h (187%) in the presence of the GGMA/TA-based patch (dotted bar in Figure 4A and Figure 4B, respectively), compared to the control cells (white bar), suggesting that the GGMA/TA combination influences the percentage of metabolically active cells, which is directly correlated to cell viability, as already demonstrated elsewhere [49]. Furthermore, a significant increase in HDF cell viability with a GGMA-based patch at 48 h (177%) was observed (black bar of Figure 4B), compared to the untreated cells (white bar). These results confirm that the GGMA patch alone is able to promote cell viability, probably due to its porous, organized 3D structure, which mimics the biological and mechanical features of the natural tissue [50].

Consequently, the indirect cytotoxicity test was performed on HDF cells treated for 24 and 48 h with GGMA, GGMA/TA and GGMA/AA-patch-conditioned media. The HDFs maintained in a non-conditioned medium were used as control/untreated cells. Figure 4C,D show no significant results for any condition compared to the control. However, even if the decrease in viability in cells treated with GGMA/TA-patch-conditioned medium in the indirect test is not significant when compared to the HDFs with GGMA/TA patch conditioning in the direct test, it seems to have an opposite trend. In the direct test, the cell viability appeared further enhanced by the TA addition to the GGMA patches, although it decreased in the indirect test, suggesting that the controlled-manner release of the TA has a different influence on HDFs with respect to their high-concentration (all-in-one) stimulation. Similar TA behavior has been already suggested in a study by Kaczmarek et al. [49], where it was suggested that in materials composed of chitosan and TA, their ratio may influence the percentage of metabolically active cells. On the other hand, the result of HDFs with the GGMA/AA patch in the direct test and that of HDFs with GGMA/AA-patch-conditioned medium in the indirect test appear to have different behavior; i.e., the cell viability seems to slightly decrease in the direct test and slightly increase in the indirect test. Like for the TA-loaded patches, the AA-loaded ones also showed discordant behavior in the direct and indirect tests on HDF, suggesting different bioactive properties of AA based on the exposure of HDF to its different concentrations (controlled vs. all-in-one stimulus). It is conceivable that different concentrations of AA might exert different kinds of functions in wound healing activity, as suggested elsewhere. Indeed, it was found that at lower concentrations (10–20 μg/mL) the fibroblast migration was increased, while at a higher concentration (50 μg/mL), even if the migration was decreased, the secretion of pro-wound healing factors was enhanced [51]. This property of the AA should be certainly further investigated in reference to the AA-loaded patches for wound dressing approaches.

Finally, to investigate the effect of the culture medium conditioned with the three synthesized patches (GGMA, GGMA/AA and GGMA/TA) on HDFs’ migration rate, a wound healing assay was performed.

**Figure 5:**
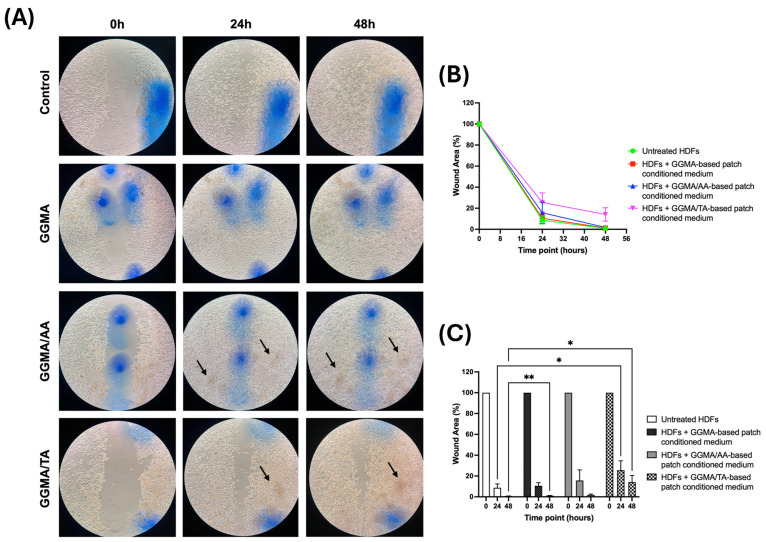
(**A**) Representative optical microscope images showing the area covered by the cells at 0, 24 and 48 h after wounding in the four different conditions (magnification 10×). Black arrows indicate cell aggregates. (**B**) Time course curves of the wound healing assay: control, i.e., untreated cells, green line; GGMA-based patch-conditioned medium, red line; GGMA/AA-based patch-conditioned medium, blue line, GGMA/TA-based patch-conditioned medium, magenta line; (**C**) Histograms of the wound healing assay: control, i.e., untreated cells, first bars; GGMA-based patch-conditioned medium, second bars; GGMA/AA-based patch-conditioned medium, third bars, GGMA/TA-based patch-conditioned medium, fourth bars. Data are reported as means ± S.D., (two-way ANOVA followed by Bonferroni’s test). * *p* < 0.012; ** *p* < 0.0017.

The obtained results (Figure 5) show that when treated with the GGMA-patch-conditioned medium (second line of microscope images in Figure 5A; red line in Figure 5B; black bars in Figure 5C), the HDF cells efficaciously migrate, healing the wound similarly to the control/untreated cells (first line of microscope images in Figure 5A; green line in Figure 5B; white bars in Figure 5C), both at 24 and 48 h; however, a statistical significance at 48 h was observed, suggesting a major migratory ability of untreated cells. Furthermore, comparing the migrating rate of control HDFs with HFD cells treated with GGMA/TA-patch-conditioned medium (fourth line of microscope images in Figure 5A; magenta line in Figure 5B; dotted bars in Figure 5C) a significance was detected at 24 and 48 h, indicating a delayed migration rate of treated cells resulting in a cell-free area of ~25% at 24 h after the wounding, and incomplete healing after 48 h. No significant results have been obtained by the cells treated with the GGMA/AA-patch-conditioned medium (third line of microscope images in Figure 5A; blue line in Figure 5B; grey bars in Figure 5C); however, the trend of migration seems similar to that of control cells and GGMA-patch-treated cells.

Altogether, these results suggest that the presence of GGMA-based patches, alone or coupled with TA or AA, may induce a different, or opposite, behavior of human dermal fibroblasts. We argue that both the time of contact of GGMA-based patches with cells and the dose- and time-dependent release of acids from patches are essential for the patches to perform at their maximum potential. For example, the direct biocompatibility test, where we use the patch in direct contact with cells, seems to show a lower potential of GGMA-based patches at 24 h, instead of 48 h; it seems to induce greater cell viability than control. Instead, observing the indirect biocompatibility test, where we use the conditioned medium, the results appear to be opposite to the direct test. The behavior could be explained assuming that the conditioned medium does not follow the gradual (dose- and time-dependent) release of the acids from the patch, but on the contrary, the conditioned medium represents the maximum concentration of acid released by the patch. We assume that the contact of the HDF cells directly with the maximum concentration determines cell stress that induces cell mortality or delays the cell cycle. This result is also supported by the wound healing assay where, following the same principle of the indirect test, the presence of acids does not lead to a faster wound closure if compared to the control, but rather, we observe a delay in wound healing or a lack of wound closure at 48 h in the GGMA/TA-conditioned medium. Finally, when the optical microscope images are observed, the cellular densities shown by the dark areas (indicated by black arrows), even in the presence of media-rich ascorbic or tannic acid, suggest quite a good cellular presence (living or no longer living cells). However, the lack of a patch could not determine the correct orientation of cell migration. In conclusion, these results suggest that the complex biomaterial/bioactive compound, determining the controlled molecule release and progressive stimulation of the surrounding cells, allows the exertion of different activities. Thus, further studies should be conducted in this regard to maximize the patches’ benefits.

### 2.4. Antimicrobial Properties

Among the factors preventing wound healing is bacterial biofilm, a slime produced by a bacterial colony to evade human defenses and promote bacterial growth. Biofilm can create a wound environment with low pH and O_2_ levels. Additionally, this film can form a physical barrier that stops cellular movement and the penetration of antibodies and antibiotics [2]. *Pseudomonas aeruginosa*, *Staphylococcus aureus*, *Klebsiella pneumoniae*, *Enterococcus faecalis* and *Acinetobacter baumannii* are among the most frequent bacterial species that cause wound infections posing a threat to human health. Gram-positive bacteria, particularly *S. aureus,* seem to be the most common colonizers, especially during the first week of an infection. Gram-negative bacteria, such as *P. aeruginosa* and *A. baumannii*, or *Escherichia coli*, begin colonizing the site at the start of the second week and can cause sepsis if they enter the blood vessels and lymphatic system [52]. In recent years, polymers modified with biocidal substances have been widely developed. Furthermore, TA and AA are known for their antimicrobial properties [53,54].

**Figure 6:**
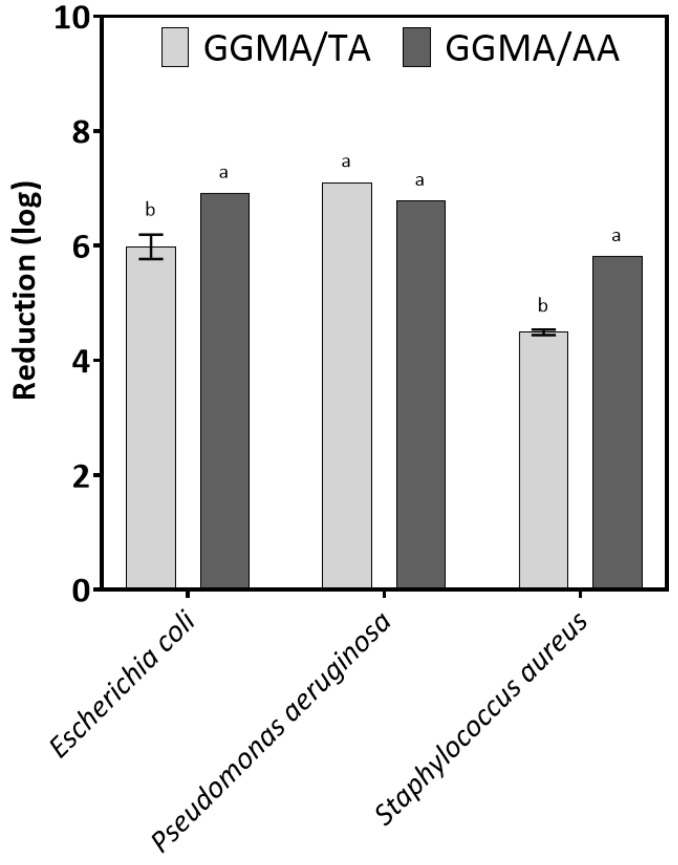
Antimicrobial activity of films against tested bacteria. Statistical significance was tested within a pathogen. Letters on the bars of the figure indicate statistically significant differences (*p* < 0.05). Statistical analysis was performed using Past v. 4.15. ANOVA and Tukey’s post-hoc test were applied.

The antimicrobial properties of the tested materials are shown in Figure 6. The results from antimicrobial tests highlighted that GGMA modified with TA and AA had biocidal activity against the three tested bacteria, *E. coli*, *P. aeruginosa* and *S. aureus*, used as models. The reduction number of strains was over 3 log, indicating biocidal properties. The material GGMA/TA had a weaker biocidal effect against *E. coli* and *S. auresus* than GGMA/AA. However, the biocidal properties of the tested materials against *P. aeruginosa* were similar. Different films based on derivatives of GGMA have been modified with various biocidal compounds, and the data showed that their antimicrobial activity depends on the concentration of the biocide. Often, the minimum biocidal concentration (MBC) of the biocide is lower than when it is incorporated into the polymer. Therefore, it is important to look for biocides that are effective at low concentrations so as not to cause cytotoxic effects. For example, Di Muzio et al. [55] developed nanocomposite films based on GGMA modified with silver nanoparticles (AgNPs), showing that the nanocomposite was able to inhibit the growth of *E. coli* and *S. aureus*. Pacelli et al. considered that antimicrobial agents need to be introduced to achieve good properties [56]. Hua et al. realized a GG-based coating on 3D printed porous tantalum scaffolds, crosslinked with both isoniazid and rifampicin as drugs [57]. The obtained coatings demonstrated significant bactericidal effects against *S. aureus*, highlighting that the simultaneous release of different biocides from coatings is more efficient than the release of one biocide alone [58]. In conclusion, our results open prospects for using GG derivatives as bioactive scaffolds with antibacterial properties. Tannic acid and ascorbic acid incorporated into GG derivatives appear to be better biocides than, for example, antibiotics, which can lead to problems of antibiotic resistance.

## 3. Conclusions

Wound healing is a complex process that often requires multifunctional 3D smart wound dressing. Therefore, in the present study, bioprinting was employed to successfully design and develop bioactivated methacrylated gellan gum (GGMA) patches incorporating tannic acid (TA) and L-ascorbic acid (AA), aimed at promoting healing after extensive skin tissue defects. The combination of suitable physicochemical (i.e., swelling behavior and controlled bioactive molecule release), mechanical and morphological properties allowed obtaining patches addressing the critical needs in wound care management. All patches demonstrated excellent cytocompatibility with human dermal fibroblasts, promoting cell proliferation and overall wound healing efficiency. Furthermore, they highlighted biocidal activity against the three tested bacteria, making them suitable for use as advanced multifunctional dressings.

## 4. Materials and Methods

### 4.1. Synthesis and Characterization of Photocrosslinkable GGMA

Low-acyl gellan gum (GG, Gelzan, Sigma Aldrich, 1 g) was solubilized at 90 °C in distilled water (diH_2_O, 100 mL) under constant stirring. The material was functionalized by a reaction with methacrylic anhydride (MA, purity ≥ 94%, Sigma Aldrich) for 6 h at 50 °C, as described in a previous paper [8]. A 20 mol % MA excess per unit of GG was employed. In addition, 5M sodium hydroxide (NaOH, reagent grade, 98%, Sigma Aldrich) was added dropwise to maintain the pH between 8 and 8.4. Afterward, the GGMA was precipitated under stirring in cold absolute ethanol (purity ≥ 99.9%, Sigma Aldrich) using a volume of EtOH 4 times bigger than the GGMA solution. GGMA was solubilized in diH_2_O and purified by dialysis (12–14 kDa molecular weight cut-off; Sigma Aldrich, Milan, Italy) in deionized water (dH_2_O, LC-MS grade, conductance at 25 °C ≤ 1 µS/cm, Sigma Aldrich, Milan, Italy) for 72 h. The material was lyophilized (LaboGene’s CoolSafe 55-4 PRO, Bjarkesvej, Denmark) and stored at −80 °C for further use.

### 4.2. Preparation of GGMA Patches

**Figure 7:**
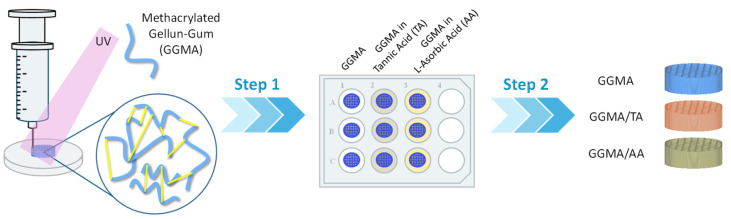
Representative scheme of producing 3D methacrylated gellan gum (GGMA), GGMA/tannic acid (TA) and GGMA/L-ascorbic acid (AA) patches, from 3D bioprinting to TA and AA loading.

GGMA patches were produced according to the scheme reported in Figure 7. The optimization of the printing conditions is reported in previous studies [8]. GGMA (4% *w*/*v*) was solubilized in diH_2_O at 70 °C for 2 h. Afterward, 2-hydroxy-4′-(2-hydroxyethoxy)-2-methylpropiophenone (Irgacure 2959, gas chromatography area ≥ 98%, Sigma Aldrich) at 5% concentration (w_Irgacure_/w_GGMA_) was added in the dark to obtain a homogeneous ink formulation. The 3D printing was performed through “Rokit Invivo 4D2”. The printing model was sliced using NewCreatorK 1.57.70 (Rokit Healthcare Inc., Seoul, Republic of Korea). A 27-gauge needle (0.210 ± 0.019 mm nominal inner diameter; 0.4128 ± 0.0064 nominal outer diameter), 0.3 mm layer thickness and 45% infill density were used to build cylindrical patches (⌀ = 13 mm, t = 3 mm). Horizontal and vertical struts were alternated with each layer following a grid pattern with 0/90 fiber orientation. The printing process was performed at room temperature. The speed was set at 4 mm/s for a total printing time of 9 min and 45 s. After printing, the patches were chemically crosslinked by exposure to UV light (Analytik Jena UVP crosslinker, Jena, Germany, λ: 365 nm, P: 10 J/cm^2^) for 10 min. The patches were also physically crosslinked by immersion in 0.05% *w*/*v* CaCl_2_ at room temperature.

L-ascorbic acid (LA, 99%, Sigma Aldrich, Milan, Italy) and tannic acid (TA, ACS regent, Sigma Aldrich, Milan, Italy) were loaded into the patches by the diffusion filling method. A volume of 500 μL from 4 mg/mL of AA and TA (2 mg each patch) was loaded into 20 mg GGMA patches (pH 7.4), in agreement with different works reported in the literature [30,47,59]. After washing three times with diH_2_O, the patches were freeze-dried and stored at −80 °C.

### 4.3. Characterization of GGMA Patches

#### 4.3.1. Physicochemical Characterization

To assess the GG functionalization, Fourier transform infrared (ATR-FTIR) spectroscopy (Thermo Fisher Nicolet IS10, Waltham, MA, United States of America) was employed. GGMA was scanned from 650 to 4000 cm^−1^ with a resolution of 2 cm^−1^. Similarly, GGMA/AA and GGMA/TA patches were analyzed. Neat GG, AA and TA powders, under the same conditions, were used as control.

#### 4.3.2. Morphological Analysis

3D bioprinted patches were observed by scanning electron microscopy (SEM, FEI Quanta 200 FEG, Hillsboro, OR, United States of America). Patches were washed with dH_2_O, frozen at −80 °C and lyophilized. Subsequently, after being coated with an ultrathin layer of Au/Pt using an ion sputter, dried samples were observed by SEM.

#### 4.3.3. Mechanical Analysis

Dynamical mechanical analysis (DMA, TA-Q800, TA-Instrument, New Castle, DE, United States of America) was used to examine bulk patches with dimensions of 8 mm in diameter and 4 mm in thickness. A force track of 125%, a preload of 0.001 N, an amplitude of 100 µm in compression and a frequency of 1.0 Hz were set. The tests were conducted at ambient temperature and in a confined environment in wet conditions.

#### 4.3.4. Swelling Studies

After being weighed (*w*_0_), lyophilized scaffolds were allowed to swell in sterile Dulbecco’s Modified Eagle Medium, high-glucose, without phenol red (DMEM-HG, Gibco by Thermo Fisher Scientific, Monza, Italy) up to 28 days (pH 7.4, T = 37 °C, V = 5 mL). Antibiotics were added to DMEM-HG to replicate physiological conditions. The swollen hydrogels were then removed at predetermined intervals, the superficial adsorbed solution was rapidly removed with a paper, the weight (*w_t_*) was noted and the samples were once more immersed in the medium to evaluate their swelling capability. The swelling ratio (*Q*) was calculated according to Equation (1):

(1) $$Q=\frac{w_{t}-w_{0}}{w_{0}}$$

where *w*_0_ indicates the weight of the dried sample before immersion.

#### 4.3.5. Release Studies

All measurements of AA and TA from GGMA/AA and GGMA/TA patches were performed using a UV spectrophotometer (SPECORD 210 PLUS Double-beam Spectrophotometer, Analytik Jena, Jena, Germany) at an absorbance wavelength of 252 nm and 275 nm, respectively [39,40]. Eight concentrations of ascorbic acid and tannic acid freshly prepared in dH_2_O, respectively, were first immediately measured to create a calibration curve. The calibration curve was linear with a correlation coefficient of R^2^ = 0.9984 (y = 56.269x + 0.0838) for AA and of R^2^ = 0.9997 (y = 45.161x + 0.0149) for TA, and the lower limit of detection was 0.05 mg/mL for both agents. Three patches for each group were placed into 5 mL of dH_2_O, in a dry incubator at 37 °C. A vehicle patch was taken as a control. At 1, 2, 4, 6, 24, 48 and 72 h and 6, 12 and 20 days, the absorbance was read. The concentration was determined using the calibration curve. Experiments were repeated three times.

#### 4.3.6. Antioxidant Test

The 2,2-diphenyl-1-picrylhydrazyl (DPPH, Sigma Aldrich, Milan, Italy) radical scavenging method was employed to assess the antioxidant activity of GGMA, GGMA/AA and GGMA/TA patches by using the protocol of Huang et al. [30]. The DPPH solution (39.5 mg/L) was prepared by dissolving DPPH in EtOH/diH_2_O 1:1 *v*/*v*. Then, each sample was immersed in 3 mL DPPH solution, in a dark environment for 1.5 h. Changes in absorbance at 517 nm were measured using UV-VIS. The DPPH radical scavenging activity (SA_DPPH_) was calculated according to the following Equation (2):

SA_DPPH_ (%) = (A_blank_ − A_sample_)/A_blank_ × 100%, (2)

where A_blank_ and A_sample_ are the absorbances of DPPH solution and samples mixed with DPPH solution, respectively. Three parallel samples were used for each sample.

### 4.4. Biological Characterization

#### 4.4.1. Cell Culture

Human dermal fibroblasts (HDFs, Gibco-Invitrogen cell culture, C-013-5C) were cultured in T25 tissue culture flasks with Dulbecco’s Modified Eagle Medium (DMEM, Sigma-Aldrich D6546) supplemented with 10% fetal bovine serum (FBS) (GIBCO Invitrogen, Milan, Italy), 100 U/mL penicillin, 100 U/mL streptomycin and 2 mM L-glutamine and incubated at 37 °C in a humidified atmosphere containing 5% CO_2_.

#### 4.4.2. Biocompatibility Direct Test According to ISO 10993-5

**Figure 8:**
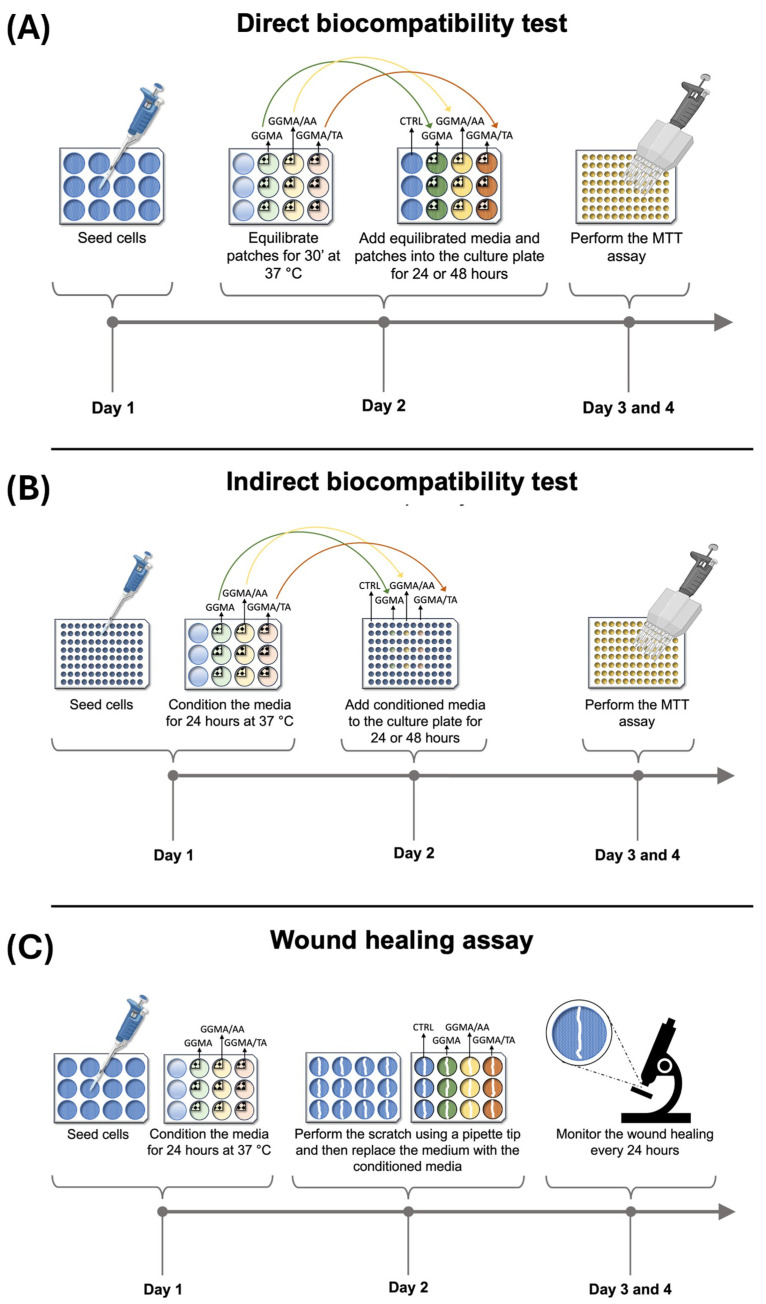
**(A**) Timeline showing the steps/day of the biocompatibility (direct) test. (**B**) Timeline showing the steps/day of the indirect test. (**C**) Timeline showing the steps/day of the wound healing assay.

To evaluate the biological safety of the three synthetized patches (GGMA, GGMA/TA and GGMA/AA) according to ISO 10993-5 standards [48], a direct biocompatibility test was performed for 24 and 48 h as shown in Figure 8A and described below. On day 1, a total of 10 × 104 HDF cells/well were seeded in two 12-well plates with 1 mL/well of complete medium. The plates were incubated 24 h at 37 °C in a humidified atmosphere containing 5% CO_2_. Two patches for each type were then cut into four pieces according to the size specifications recommended by the ISO 10993-5 guidelines [48]. On the second day, each quarter of the patches were placed in a 12-well plate and in 2 mL of complete medium for 30 min at 37 °C. At the end of the 24 h incubation time, after cell attachment, the culture medium was replaced with the medium used to equilibrate the patches, and each quarter of the patches was placed above the cells (three wells for each ¼ of the three types of patches + 3 control wells). In the three control wells, fresh complete medium was added. Then, the 12-well plates were incubated for 24 h and 48 h at 37 °C in a humidified atmosphere containing 5% CO_2_. At the end of the incubation time, an MTT assay was performed to assess cell viability.

*Direct test—MTT assay:* After 24 or 48 h, the medium was discarded and replaced with 500 μL/well of MTT solution, and the plate was incubated for 2 h at 37 °C in a humidified atmosphere containing 5% CO_2_. At the end of the incubation, the MTT solution was discarded and replaced with 500 μL/well of DMSO, and the formazan crystals were dissolved by gentle pipetting. Then, the volume of each well (500 μL) was added to 5 wells of a 96-well plate (100 μL/well), and the absorbance was measured with a microplate reader (Biochrom EZ Read 400 Microplate Reader, 22 Cambridge Science Park, Milton Road, Cambridge CB4 0FJ, UK) at 570 nm.

*Direct test—statistical analysis:* The statistical analysis was performed using GraphPad Prism 9 Software. A two-way ANOVA followed by Bonferroni’s test was performed. Data as mean ± standard deviation (S.D.) were normalized to the control condition mean.

#### 4.4.3. Biocompatibility Indirect Test According to ISO 10993-5

To evaluate the cytotoxicity of the medium conditioned with the three synthetized patches (GGMA, GGMA/AA and GGMA/TA), an indirect test was performed for 24 and 48 h as depicted in Figure 8B and described below. On day 1, a total of 1 × 10^4^ HDF cells/well were seeded in 12 wells of two 96-well plates with 200 μL/well of complete medium. The plates were then incubated for 24 h at 37 °C in a humidified atmosphere containing 5% CO_2_. On the second day, after cell attachment, the culture medium was discarded and replaced with a starvation medium (DMEM supplemented with 100 U/mL penicillin, 100 U/mL streptomycin and 2 mM L-glutamine), and the plates were incubated at 37 °C in a humidified atmosphere containing 5% CO_2_. On the same day, a patch of each of the three types (GGMA, GGMA/TA, GGMA/AA) was cut in four pieces, and each quarter was placed in a well of a 12-well plate containing 2 mL of complete medium for 24 h at 37 °C. In this manner, three different conditioned media (for each patch) were obtained. On the third day, the starvation medium was discarded and replaced with the conditioned media (three wells for each conditioned medium), and the 96-well plates were incubated for 24 h and 48 h at 37 °C in a humidified atmosphere containing 5% CO_2_. For the control wells, the starvation medium was replaced with a fresh complete medium. At the end of the incubation time, an MTT assay was conducted to assess cell viability.

*Indirect test—MTT assay*: After 24 or 48 h, the medium was discarded and replaced with 100 μL/well of MTT solution, and the plate was incubated for 2 h at 37 °C in a humidified atmosphere containing 5% CO_2_. At the end of the incubation, the MTT solution was discarded and replaced with 100 μL/well of DMSO. The formazan crystals were dissolved by gentle pipetting, and the absorbance was measured with a plate reader (Biochrom EZ Read 400 Microplate Reader, 22 Cambridge Science Park, Milton Road, Cambridge CB4 0FJ, UK) at 570 nm.

*Indirect test—statistical analysis:* The statistical analysis was performed using GraphPad Prism 9 Software. A one-way ANOVA followed by Bonferroni’s test was performed. Data as mean ± S.D. were normalized to the control condition mean.

#### 4.4.4. Wound Healing Assay

To investigate the effect of the medium conditioned with the three synthesized patches (GGMA, GGMA/AA and GGMA/TA) on cell migration rate, a wound healing assay was performed as shown in Figure 8C and summarized below.

On day 1, a total of 10 × 10^4^ HDF cells/well were seeded in a 12-well plate with 1 mL of complete medium and incubated for 24 h at 37 °C in a humidified atmosphere containing 5% CO_2_. On the second day, after cell attachment, a wound was created at the center of each well by scratching the confluent cell monolayer with a 200 μL pipette tip. After the scratch, the medium of each well was replaced with the medium conditioned for 24 h with each of the three patches as previously described (three wells for each conditioned medium). In the three control wells, the medium was replaced with fresh complete medium. The wound healing was monitored with a phase-contrast microscope (MARCA) and by taking photos every 24 h from scratch. The assay was stopped after 48 h since the heal appeared closed in the control wells and the wells containing the medium conditioned with the GGMA patch.

*Wound healing assay—statistical analysis:* The statistical analysis was performed using GraphPad Prism 9 Software. A two-way ANOVA followed by Bonferroni’s test was performed. Data were reported as mean ± S.D., compared to the control condition mean.

### 4.5. Antimicrobial Properties

The antimicrobial properties of the materials were evaluated against the pathogens *Pseudomonas aeruginosa* ATCC 15442, *Staphylococcus aureus* ATCC 6538 and *Escherichia coli* ATCC 8739 in accordance with the ISO 22196:2011 standard [60], which corresponds to 1.5 × 10^8^ bacterial cells per 1 mL according to the McFarland scale. The test materials were then placed in a sterile dish, and 0.2 mL of bacterial suspension was applied. To maintain a humid environment, a piece of sterile filter paper soaked with 2 mL of sterile diH_2_O was placed in the dish, ensuring it did not touch the materials. GGMA was treated as a control sample. The materials were incubated at 37 °C for 24 h. After incubation, the number of bacterial cells on both the tested and control materials was determined. To recover the bacteria from the surface of the materials, the materials were placed in 10 mL of neutralizer medium and shaken for 3 min. A series of dilutions were then prepared, and the bacteria were inoculated by the pour plate method on PCA medium (Biomaxima, Lublin, Poland) and incubated for 24 h at 37 °C. The JIS Z 2801:2010 Standard [61] was used to compute the decrease in the quantity of bacteria. It is represented by an R-value. The viable bacteria cell number in the control sample (PVA) after 24 h of incubation (CFU/mL) is denoted by B, and the viable bacteria cell number in the test samples (PVA/STO) after 24 h of incubation (CFU/mL) is denoted by C. The R-value is equal to log (B/C). After 24 h, GGMA/AA and GGMA/TA were the averages of the common logarithm of the number of live bacteria recovered from the test samples. The criteria employed by Souli et al. [62] were utilized to assess the antimicrobial activity’s efficacy. These criteria included a reduction in suspension density that ranged from ≤2 to <3 log mean bacteriostatic characteristics and a reduction of more than 3 log bactericidal features.

## Data Availability

The authors declare that all data supporting the findings of this study are available within the paper; source data for the figures in this study are available from the authors upon request.

## Supplementary Materials

The following supporting information can be downloaded at https://www.mdpi.com/article/10.3390/gels11010040/s1: Figure S1. Optical images of the patches during the different steps of the production: (a) during 3D printing, (b) after 3D printing, (c) after crosslinking by exposure to UV light for 10 min and dipping in 0.05% w/v CaCl_2_ at room temperature, (d) after freeze-drying and (e) after tannic acid (TA) and L-ascorbic acid (AA) diffusion filling into methacrylated gellan gum patches (GGMA).
