# Supplementary material for: Exploring Methacrylated Gellan Gum 3D Bioprinted Patches Loaded with Tannic Acid or L-Ascorbic Acid as Potential Platform for Wound Dressing Application
Source: Gels. 2025 Jan 5;11(1):40. doi: 10.3390/gels11010040 (PMC11765158; doi:10.3390/gels11010040)
Supplement: Supplementary file 1 [file gels-11-00040-s001.zip › gels-3370289-supplementary.pdf]

Supplementary Materials

Exploring Methacrylated Gellan Gum 3D Bioprinted Patches Loaded with Tannic Acid or L-Ascorbic Acid as Potential Platform for Wound Dressing Application

Federica Scalia [1,†], Alessandra Maria Vitale [1,†], Domiziana Picone [1], Noemi De Cesare [2], Maria Swiontek Brzezinska [3], Beata Kaczmarek-Szczepanska [4], Alfredo Ronca [2], Barbara Zavan [5], Fabio Bucchieri [1], Marta Anna Szychlinska [6,‡] and Ugo D'Amora [2,*,‡]

[1] Department of Biomedicine, Neuroscience and Advanced Diagnostics (BIND), University of Palermo, 90127 Palermo, Italy; federica.scalia02@unipa.it (F.S.); alessandramaria.vitale@unipa.it (A.M.V.); domiziana.picone@unipa.it (D.P.); fabio.bucchieri@unipa.it (F.B.)
[2] Institute of Polymers, Composites and Biomaterials (IPCB), National Research Council (CNR), 80125 Naples, Italy; noemidecesare@cnr.it (N.D.C.); alfredo.ronca@cnr.it (A.R.)
[3] Department of Environmental Microbiology and Biotechnology, Faculty of Biological and Veterinary Sciences, Nicolaus Copernicus University in Torun, Lwowska 1, 87-100 Torun, Poland; swiontek@umk.pl
[4] Department of Biomaterials and Cosmetics Chemistry, Faculty of Chemistry, Nicolaus Copernicus University in Torun, Gagarin 7, 87-100 Torun, Poland; beata.kaczmarek@umk.pl
[5] Department of Translational Medicine, University of Ferrara, 44121, Ferrara, Italy; barbara.zavan@unife.it
[6] Department of Precision Medicine in Medical, Surgical and Critical Care (MEPRECC), University of Palermo, 90127 Palermo, Italy; martaanna.szychlinska@unipa.it
* Correspondence: ugo.damora@cnr.it
† These authors contributed equally to the manuscript.
‡ These authors contributed equally to the manuscript.

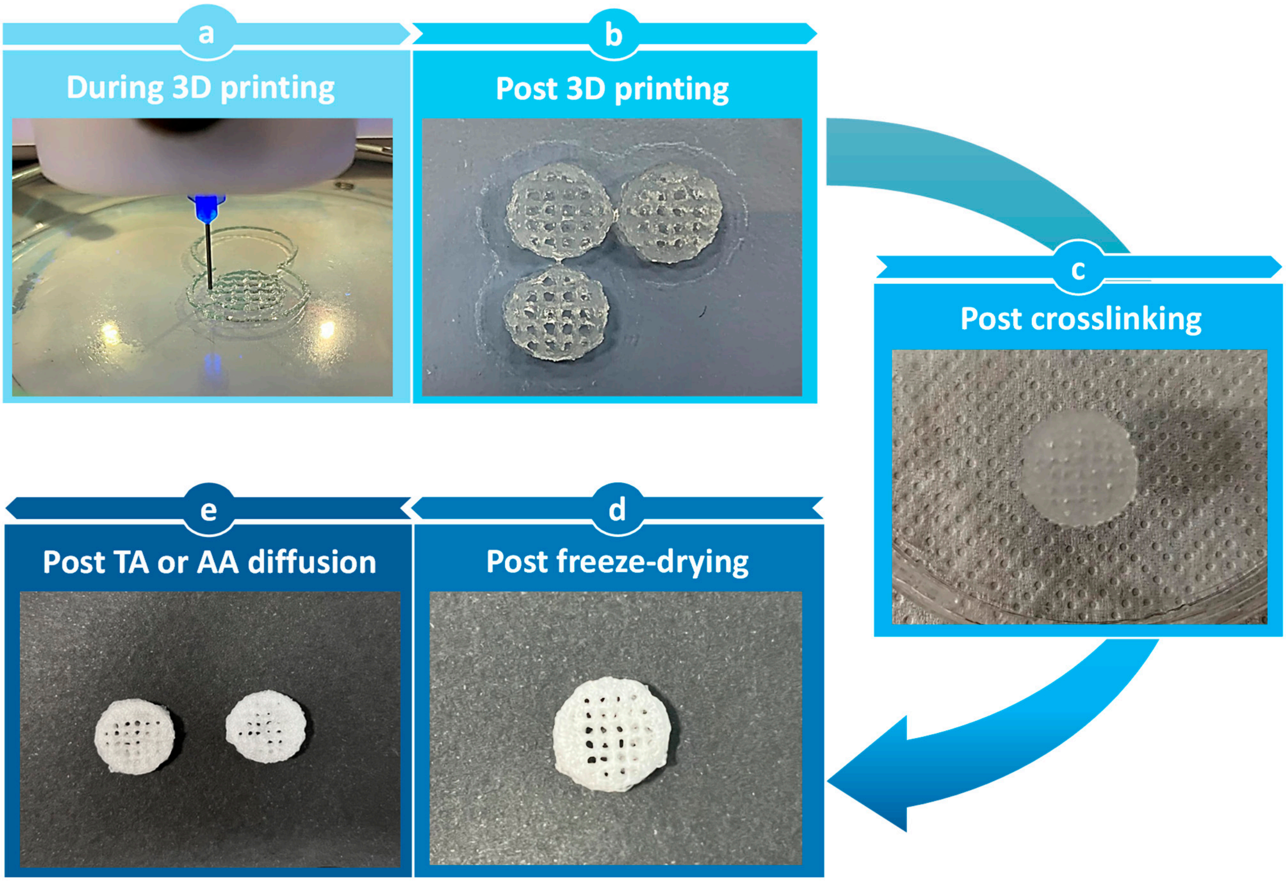

Figure S1. Optical images of the patches during the different steps of the production: (a) during 3D printing, (b) post 3D printing, (c) post crosslinking by exposure to UV light for 10 min and dipping in 0.05% w/v $CaCl_2$ at room temperature; (d) post freeze-drying and (e) post tannic acid (TA) and L-ascorbic acid (AA) diffusion filling into methacrylated gellan gum patches (GGMA).
